# Microbiological Investigations for Chikungunya Virus in Children With Acute Encephalitis Syndrome in a Non‐Outbreak Setting in Southern India

**DOI:** 10.1002/jmv.70233

**Published:** 2025-02-15

**Authors:** Tina Damodar, Chitra Pattabiraman, Bhagteshwar Singh, Maria Jose, Namratha Prabhu, Akhila L, Pramada Prasad, Uddhava V. Kinhal, A. V. Lalitha, Fulton Sebastian Dsouza, Sushma Veeranna Sajjan, Vykuntaraju K. Gowda, Vasanthapuram Ravi, Ruwanthi Kolamunnage‐Dona, Benedict D. Michael, Tom Solomon, Ravi Yadav, Lance Turtle

**Affiliations:** ^1^ Department of Neurovirology National Institute of Mental Health & Neurosciences Bangalore India; ^2^ Tropical & Infectious Diseases Unit Royal Liverpool University Hospital Liverpool UK; ^3^ Institute of Infection, Veterinary and Ecological Sciences University of Liverpool Liverpool UK; ^4^ Department of Infectious Diseases Christian Medical College Vellore India; ^5^ Department of Pediatric Neurology Indira Gandhi Institute of Child Health Bangalore India; ^6^ Department of Pediatric Critical Care St John's Medical College and Hospital Bangalore India; ^7^ Department of Pediatrics St John's Medical College and Hospital Bangalore India; ^8^ Department of Pediatrics Bangalore Medical College and Research Institute Bangalore India; ^9^ Department of Health Data Science, Institute of Population Health University of Liverpool UK; ^10^ Department of Clinical Infection Microbiology and Immunology, Institute of Infection, Veterinary, and Ecological Science University of Liverpool Liverpool UK; ^11^ National Institute for Health and Care Research Health Protection Research Unit in Emerging and Zoonotic Infections University of Liverpool Liverpool UK; ^12^ The Pandemic Institute Liverpool UK; ^13^ Department of Neurology The Walton Centre NHS Foundation Trust Liverpool UK; ^14^ Department of Neurology National Institute of Mental Health & Neurosciences Bangalore India; ^15^ National Institute for Health and Care Research Health Protection Research Unit in Emerging and Zoonotic Infections, Institute of Infection, Veterinary and Ecological Sciences University of Liverpool; ^16^ Liverpool University Hospitals NHS Foundation Trust Liverpool UK

## Abstract

Chikungunya virus (CHIKV) is an emerging cause of acute encephalitis syndrome (AES) in India, with limited data on its role in childhood AES in southern India. We systematically evaluated children with AES in southern India during a non‐epidemic period for CHIKV. Serum and cerebrospinal fluid (CSF) samples were tested for CHIKV using IgM ELISA and real‐time reverse transcriptase PCR. Amplicon sequencing was performed on PCR‐positive samples. Clinical and laboratory features were compared between children with and without CSF CHIKV positivity (PCR/IgM antibodies). Of 376 children with AES, 20 (5.3%) had positive CHIKV tests. Co‐infections were common, particularly with scrub typhus. Children presented with diverse symptoms affecting various organ systems. Neurological manifestations included meningism, seizures, cerebellar signs, behavioral abnormalities, cranial nerve involvement, involuntary movements, and hemiparesis/hemiplegia. Children with CSF CHIKV positivity showed more focal neurological deficits and transaminitis, and less musculoskeletal symptoms. Sequencing confirmation of CHIKV was made in all patients with positive CHIKV PCR, revealing a close relationship with 2016 Kenyan and Indian strains, albeit in a different clade within the East/Central/South African genotype. Along with important mutations known to impact CHIKV infectivity, four novel amino acid substitutions were detected in envelope protein coding regions. Our findings underscore the importance of routine and comprehensive CHIKV testing for children with AES, irrespective of season/outbreak. The high rate of co‐infections warrants further research. Continued genomic surveillance is essential to monitor emerging mutations with epidemic potential, increased severity and the risk of neurological disease.

## 1 Introduction

Chikungunya virus (CHIKV), an alphavirus within the family *Togaviridae*, is primarily transmitted between humans by mosquitoes of the genus *Aedes* [1]. In adults, CHIKV infection typically manifests with fever and severe joint pain, while children experience fewer musculoskeletal symptoms but are more likely to be hospitalized due to neurological complications, including encephalitis, meningoencephalitis, seizures, and other neurological disorders [2, 3, 4].

CHIKV‐associated neurological disease was first reported during an Asian strain outbreak in 1964 in Chennai, southern India [3, 5]. During the 2005 La Réunion epidemic, linked to the East/Central/South African (ECSA) strain, neurological cases were found to be more common [6]. Subsequently, neurological manifestations have been documented in areas affected by both ECSA and Asian strains worldwide [3, 7, 8, 9, 10, 11, 12, 13, 14]. Although mutations within the CHIKV genome can lead to increased vector adaptability and infectivity, their association with neurovirulence remains uncertain [15, 16].

India experienced recurrent chikungunya outbreaks between 2005 and 2019, establishing nationwide endemicity and IgG seroprevalence of 11.2% in children in southern India [1, 17]. Studies predominantly in adults report neurological manifestations in a wide range, 9%–47%, of hospitalized patients during CHIKV outbreaks in India [9, 18, 19, 20, 21, 22]. While data on the pediatric population in India are lacking, studies from other countries also report a wide prevalence of neurological symptoms in children between 4.7% and 46% during outbreaks [3, 6, 8, 10, 11, 21]. A recent large‐scale study conducting comprehensive surveillance of acute encephalitis syndrome (AES), a major public health concern in India predominantly affecting children, found CHIKV in 3% of children with AES in northern India during the 2017 chikungunya outbreak, highlighting the need for enhanced testing of AES patients for endemic pathogens beyond those commonly considered, such as Japanese encephalitis virus (JEV) [23].

AES is broadly defined to include cases with fever and altered mental state, which may result from encephalitis (brain inflammation due to direct central nervous system [CNS] involvement by pathogen) or encephalopathy resulting from severe systemic disease [24, 25]. Differentiating between CHIKV‐associated encephalitis and encephalopathy is crucial for appropriate management and prognosis. Encephalitis can lead to more severe disease requiring intensive care [26, 27]. Clinical diagnosis is challenging due to overlapping clinical features with other tropical infections and therefore simultaneous microbiological evaluation is essential [25]. Detecting CHIKV RNA or IgM in cerebrospinal fluid (CSF) provides the strongest evidence of causality with encephalitis [3, 24]. Notably, unlike other arboviral encephalitides, CSF pleocytosis is not always seen in CHIKV encephalitis [9, 28].

This study presents findings of CHIKV investigation in children with AES recruited through an ongoing study in southern India, which aims to develop clinical prediction models for treatable causes of AES. During recruitment and microbiological investigations, we observed several patients with evidence of CHIKV infection, despite the absence of any ongoing chikungunya outbreak. Given the limited data on CHIKV in children with AES in India, we conducted a comprehensive study to characterize the clinical, laboratory, and genomic features of CHIKV‐associated AES in southern India during a non‐outbreak period, aiming to fill critical knowledge gaps. Our findings have important implications for improving diagnosis, management, and understanding of the role of CHIKV in pediatric AES in endemic regions.

## 2 Methods

### 2.1 Patients and Study Sites

In this prospective multicenter observational study, we recruited children aged 1 month–18 years, meeting the Indian National Vector Borne Disease Control Program and World Health Organization criteria for AES. These children were admitted to three tertiary care hospitals in Bangalore, Karnataka, southern India: Indira Gandhi Institute of Child Health, St. John's Medical College and Hospital, and Vani Vilas Hospital (Bangalore Medical College). Briefly, this study enrolled children presenting with fever or history of fever and a change in mental state, with illness duration of less than 30 days [29]. Neonates, children with immunodeficiency and those with known noninfectious illnesses were excluded from the study. The study was approved by the institutional ethics and review boards of the recruiting hospitals and the coordinating centre, the National Institute of Mental Health and Neurosciences (NIMHANS). Written consent was obtained from caregivers, and assent was sought from older children using procedures and forms approved by the institutional ethics committees. Here we present results of patients recruited between March 2020 and March 2022.

### 2.2 Microbiological Testing

Blood and CSF samples were sent to the Department of Neurovirology, NIMHANS. Samples were systematically tested for microbiological evidence of pathogens commonly associated with AES in the region, including JEV, dengue virus (DENV), CHIKV, *Orientia tsutsugamushi* (causative agent of scrub typhus), *Leptospira* sp, *Plasmodium* sp, herpes simplex virus, varicella zoster virus, mumps virus, measles virus, West Nile virus, parechovirus, *Mycobacterium tuberculosis*, *Streptococcus pneumoniae, Neisseria meningitidis*, and *Haemophilus influenzae*; using a combination of serological and molecular methods as previously described [25].

All serum samples were screened for anti‐CHIKV IgM antibodies using ELISA (Inbios International, USA). For patients positive for anti‐CHIKV IgM in serum, CSF was tested for anti‐CHIKV IgM, and both serum and CSF were tested for CHIKV RNA using real‐time reverse transcriptase PCR (RT‐PCR). Sample extraction was performed using the QIAamp Viral RNA Mini Kit (QIAGEN, Hilden, Germany), followed by TaqMan real‐time RT‐PCR targeting the nonstructural protein four region of CHIKV [30]. In addition, for any patients without a microbiological diagnosis after testing for above‐mentioned pathogens, CHIKV RT‐PCR and anti‐CHIKV IgM ELISA were performed on stored CSF samples. Finally, serum and CSF samples from patients with clinical suspicion of CHIKV infection, as indicated by the treating physician, were subjected to CHIKV RT‐PCR, regardless of the anti‐CHIKV IgM ELISA result. A cycle threshold (Ct) value less than 40 was considered positive.

Given the high rate of co‐positivity, the likely cause of CNS infection by CHIKV was determined based on microbiological test results for CHIKV and CSF analysis, as detailed in Table S1.

### 2.3 Clinical Assessment and Data Collection

Clinical, demographic, and routine laboratory results were collected at the time of hospital admission and entered online using an online platform (https://www.clappia.com) [31]. Wherever available, brain imaging (MR/CT scan) findings were also noted. With assistance from caregivers, patients were followed up by telephone 3 months after being discharged from hospital, at which point the Liverpool Outcome Score (LOS) tool was applied to assess neurological function and outcomes were scored between 1 and 5, as described previously [32]. Outcomes were classified “Good” at a score of 5 (full recovery) or 4 (minor sequelae), and “Poor” at a score of 3 (moderate sequelae), 2 (severe sequelae), or 1 (death). Normal ranges of routine laboratory tests and measurements of organ dysfunction were defined according to established criteria [33]. Clinical and laboratory features were compared between children with and without microbiological evidence of direct CNS involvement by CHIKV (based on CSF CHIKV positivity‐ CSF PCR+ or CSF anti‐CHIKV IgM+). We also compared characteristics between CHIKV‐positive and CHIKV‐negative groups (based on microbiological test results).

### 2.4 CHIKV Amplicon Sequencing‐Library Preparation and Sequencing

CHIKV amplicon sequencing was performed on samples that were positive for CHIKV by RT‐PCR. Briefly, cDNA was prepared using the LunaScript RT SuperMix Kit (New England Biolabs). Multiplex PCR primers spanning the entire CHIKV genome were designed using PrimalScheme software with NCBI Reference Sequence, NC_004162.2 as the reference sequence and additional primers were included to cover the untranslated regions. NC_004162.2 is the sequence of S27‐African prototype strain, isolated from a febrile patient, passaged in *Aedes albopictus* C6/36 cells [34]. Primer sequences are provided in Table S2. Singlepot amplicons were generated using two reactions as described previously with the Q5 high‐fidelity polymerase [35]. The resulting amplicons were pooled, and samples were multiplexed using a native barcoding kit (NBD104/NBD114, Oxford Nanopore Technologies [ONT]) and sequenced using the ligation kit (SQK‐LSK‐109, ONT) on the FLO‐MIN‐106 flow cell on the MinION MK1b sequencer (ONT).

### 2.5 Analysis of Sequence Data and Genome Assembly

Sequencing data were obtained using MinKNOW software (ONT, Oxford, UK). Sequences were basecalled, demultiplexed, and trimmed for adapters and barcodes using ont‐guppy (v6.4.6). A custom workflow was employed to recover consensus sequences of the CHIKV genomes, involving length filtering (> 100 bp) and amplicon primer trimming with fqtrim (v0.9.7). Briefly, trimmed reads were mapped to the CHIKV reference genome using minimap2 (v2.17), and alignments were examined with samtools to generate coverage tables and plots. Consensus sequences were generated using samtools (v1.17) and ivar (v1.4.2). The Chikungunya Typing Tool (v3.69) was used for genotyping the partial and complete unedited consensus sequences (1×). Complete consensus genomes were generated with a 10**×** threshold and alignment was manually inspected using gap5 (v1.2.14‐r) and AliView (v1.28). Annotations were transferred from the reference sequence. Three near‐complete genomes (> 95%) were recovered and submitted to GenBank with accession numbers OR470609‐OR470611 [OR470609 (NIMH‐CHIKV_SJ41_S), OR470610 (NIMH‐CHIKV_SJ51_S), OR470611 (NIMH‐CHIKV_SJ51_C)].

### 2.6 Phylogenetic and Mutation Analysis of Near‐Complete CHIKV Genomes

Phylogenetic analysis was conducted on near‐complete CHIKV genomes generated in this study. A total of 857 complete CHIKV genomes were downloaded from the NCBI Virus Database. These, along with sequences from the study, were classified into CHIKV genotypes using the Genome Detective Chikungunya Typing Tool (v3.69). For phylogenetic analysis, sequences were filtered to retain only the ECSA genotype, which included all three sequences from this study.

Multiple sequence alignment (MSA) was performed with MUSCLE (v3.8.425) view (v1.28), and manually inspected to remove misaligned sequences. The resulting 461 sequences of the ECSA lineage of CHIKV were used for phylogenetic analysis. Positions 77–11 313 of the reference genome (covering both coding sequences) were selected for MSA and phylogenetic analysis due to minimal missing information in this region. A maximum likelihood tree was constructed using iqtree with model testing, ultrafast bootstraps (1000 replicates), and HM045809.1 as the outgroup. The outgroup, HM045809.1 (strain LSFS), a CHIKV isolate from 1960 in the Democratic Republic of Congo, belongs to an early ECSA clade, providing a suitable reference due to its shared ancestry with circulating ECSA strains [36].

The Bayesian information criterion selected the best‐fitting model for the data, which was general time reversible with frequency (+F), invariable sites (+I), and among‐site rate variation (+G4) parameters. The consensus tree was viewed and edited in Figtree.

Regions coding for envelope proteins E1 and E2 were extracted from the MSA, translated, and compared to identify unique and shared amino acid changes based on coding sequences. The Uniprot entry Q8JUX5 was used as the reference for amino acid sequences of S27. The predicted E1 (PRO_0000226224) and E2 (PRO_0000226222) sequences were retrieved from this entry. These positions were used to define amino acid positions and reference substitutions, as commonly used in the literature [37]. However, we have also provided the CHIKV glycoprotein 2 polypeptide amino acid positions (CHIKV gp2 aa) and tabulated the equivalent translated reference sequence (NC_0004162.2) amino acid positions to enable comparisons.

### 2.7 Statistical Analysis

Statistical analysis was performed using R version 3.6.3 (The R Project for Statistical Computing). We present descriptive data for categorical variables as frequencies, percentages, or both and describe continuous variables using median and interquartile range (IQR). Continuous variables between the groups were compared using the Mann–Whitney *U* test. Categorical data were compared using a Chi‐square or Fisher's exact test, depending on sample size. A *p* value < 0.05 was considered statistically significant.

## 3 Results

A total of 423 children were referred within a 25‐month period. Of these, 20 children (4.7%) were subsequently excluded based on study criteria. Among the remaining 403, 27 (6.7%) were further excluded due to alternative diagnoses, leaving 376 patients for analysis (Figure S1).

Microbiological diagnoses were obtained in 193/376 (51%) children, while 183 (49%) had no identifiable microbiological etiology. Etiological distribution is presented in Table S3.

Microbiological test results for CHIKV were as follows—initial screening of serum samples for anti‐CHIKV IgM using ELISA yielded positive results in 19/376 (5%) children. CSF IgM ELISA and real‐time PCR were performed for these 19 children, with results detailed in Table 1.

**Table 1:** Results of microbiological test for CHIKV (*n *= 20).

Serum		CSF		
IgM	PCR	IgM	PCR	No. patients (%)
+	+	+	+	1 (5)
+	+	−	+	1 (5)
+	+	+	−	1 (5)
+	−	+	−	8 (42)
+	+	−	−	4 (21)
+	−	−	−	4 (21)
−	+	−	+	1 (5)

Additionally, one patient not residing in a JEV‐endemic district but with positive anti‐JEV IgM antibodies and negative anti‐CHIKV IgM ELISA in serum tested positive for CHIKV PCR. This additional test was performed due to clinical suspicion of an alternate etiology, because of the residency district of the patient (Table 2, S.no. 5, patient no. 15). Therefore, in total, 20/376 (5.3%) children had positive results in one or more microbiological tests for CHIKV (Table 1). No CSF samples from children without any microbiological diagnosis after testing were positive for CHIKV RNA or anti‐CHIKV IgM antibodies.

**Table 2:** Patient‐wise results of microbiological tests (*n* = 21).

		Microbiological tests for CHIKV				Microbiological tests for other pathogens					
		Blood		CSF		Blood		CSF			
S. no.	Patient no.	Serum IgM ELISA	Blood PCR	CSF IgM ELISA	CSF PCR	Serum IgM ELISA	Blood PCR	CSF IgM ELISA	CSF PCR	CSF pleocytosis	Likely cause of CNS infection (based on microbiological tests)
1	8	+	+	+	−	−	−	−	−	+	CHIKV
2	12	+	−	+	−	−		−		+	CHIKV
3	14	+	+	−	−	JEV+ dengue+	−	−	−	+	CHIKV
4	18	+	+	−	+	−	−	−	−	−	CHIKV
5	15	−	+	−	+	JEV+	−	−	−	−	CHIKV
6	21	−	−	−	+	−	−	−	−	−	CHIKV
7	2	+	+	+	+	ST+	ST+	ST+	ST+	+	CHIKV and scrub typhus
8	4	+	+	−	−	Dengue+	−	−	−	−	Unclear/CHIKV
9	13	+	+	−	−	JEV+	−	−	−	−	Unclear/CHIKV
10	17	+	−	+	−	ST+	−	−	−	−	Unclear/CHIKV
11	19	+	−	−	−	−	−	−	−	−	Unclear/CHIKV
12	1	+	−	+	−	ST+	−	ST+	ST+	+	Scrub typhus
13	9	+	−	+	−	ST+	−	ST+	ST+	+	Scrub typhus
14	16	+	−	+	−	ST+	−	ST+	ST+	+	Scrub typhus
15	20	+	−	−	−	ST+ (eschar present), JEV+, dengue+	−	−	−	+	Unclear/scrub typhus
16	3	+	+	—	−	ST+	−	ST+	−	+	Unclear
17	5	+	−	+	−	ST+, leptospira+	−	ST+	−	+	Unclear
18	6	+	−	—	−	Dengue+	−	−	−	−	Unclear
19	7	+	−	+	−	ST+	−	ST+	−	+	Unclear
20	10	+	−	+	−	ST+	−	ST+	−	+	Unclear
21	11	+	−	−	−	Dengue+	−	−	−	−	Unclear

Abbreviations: CHIKV, chikungunya virus; CNS, central nervous system; JEV, Japanese encephalitis virus; SF, cerebrospinal fluid; ST, scrub typhus.

One more child (Table 2, S.no. 6, patient no. 21), presented with AES but was later diagnosed with primary immunodeficiency disease, hence met the exclusion criteria for the main objective of the overall study. Nevertheless, this patient tested positive for CHIKV PCR in CSF, and, while this patient is not included in the cohort of 376 children, this case is presented, and phylogenetic data discussed due to the presence of confirmed CHIKV infection of the CNS.

Notably, there was microbiological evidence of other pathogens in 16 (76%) of the 21 children (Table 2). Of these 16, 5 (31%) had PCR confirmation of CHIKV; 1 had PCR confirmation for both scrub typhus and CHIKV, 7 (44%) had positive CSF anti‐CHIKV IgM ELISA; while 3 patients neither had positive CHIKV PCR or CSF anti‐CHIKV IgM ELISA (Table 2, Table S4).

Of four children with PCR‐confirmed scrub typhus, one had simultaneous PCR confirmation for CHIKV, while the other three had positive anti‐CHIKV IgM ELISA in CSF (Table 2 **)**.

While all 21 children with AES and positive microbiological tests for CHIKV had neurological manifestations at presentation, in 7 strong, evidence of CNS infection by CHIKV was present, including 1 child with convincing evidence of co‐infection with both CHIKV and scrub typhus (Table 2, S. no. 1–7). In other cases, the likely pathogen linked to CNS infection was either unclear or was an alternate pathogen, predominantly scrub typhus in our case. In all but four cases, there was either evidence of CHIKV infection of the CNS because of a positive test in CSF, or CHIKV PCR+ in blood, indicating active infection at the time of illness. In the remaining four cases, only serum IgM was positive, meaning that an alternative cause was plausible or likely.

### 3.1 Clinical, Demographic, and Routine Laboratory Findings

Among the 20 children with CHIKV included in the cohort, the male‐to‐female ratio was 3:1, with ages ranging from 2 months to 17 years (median age: 10 years, IQR: 6–13). The majority (18, 90%) were from rural districts of two southern Indian states, Karnataka and Andhra Pradesh. CHIKV positivity in AES was highest during monsoon and post‐monsoon months, with peaks in the months of July, August, and October, nevertheless, cases were reported throughout the year except between April and June (Figure S2). Most children (11, 55%) were referred from district‐level hospitals, with a median illness duration of 4 days (IQR: 3–7) before hospitalization; 14 (70%) presented within 5 days of illness onset. Samples for testing were collected between 1‐ and 9 days post‐hospitalization (median: 3 days) and at a median of 7 days (IQR: 5.8–11.2) post‐onset of symptoms.

On admission, Glasgow Coma Scale values ranged from 6 to 15 (median: 14, IQR: 11–15). The duration of hospitalization ranged from 4 to 22 days (median: 8 days, IQR: 6.5–9), with 9 (45%) children requiring intensive care. Among these, 2 (22%) had confirmed scrub typhus, and 3 (33%) had evidence of co‐infection with dengue virus. As assessed using the LOS, 9 out of 16 children (56%) with data available achieved full recovery (LOS 5/5), 5 (31%) had moderate sequelae (LOS 3/5), including 1 child PCR positive for both CHIKV and scrub typhus, and 1 each (6.25%) had minor (LOS 4/5) and severe sequelae (LOS 2/5). Clinical presentation and neurological manifestations of these children are summarized in Table S5. Brain imaging was performed in a subset of children, with the majority showing no abnormal findings (Table S6). No discernible differences were noted between children with co‐infections and those with CHIKV as the likely cause of CNS infection.

Of the 20 children, we compared clinical findings and laboratory parameters of 12 with CSF CHIKV positivity (CSF+ ) with 8 children without CSF CHIKV positivity (CSF**−**) (Tables 1 and 2). There were 10/12 (83.3%) children with focal neurological deficits in the CSF+ group compared with 3/8 (37.5) in the CSF**−** group. Musculoskeletal symptoms were more common in the CSF**−** group (4/8, 50%) than in the CSF+ group (1/12. 8.3%) (Table 3). However, given the small number of patients, these differences were not statistically significant. Transaminitis (alanine transaminase [ALT]/aspartate transaminase > 80 IU/L) was significantly more common in the CSF+ group (*p* = 0.011), with a trend towards higher ALT levels (*p* = 0.076) and more frequent hepatic dysfunction (*p* = 0.158) in the CSF+ group. CSF analysis revealed higher median total leukocyte counts (14.5 vs. 2.0 cells/µL, *p* = 0.076) and protein concentrations (49.2 vs. 24.0 mg/dL, *p* = 0.054) in the CSF+ group. The CSF+ group had a longer duration of hospitalization and more frequent intensive care management compared to the CSF**−** group, but these differences were not statistically significant.

**Table 3:** Difference between clinical and laboratory variables of children with and without CSF CHIKV positivity.

Clinical/laboratory variables	No patients (%)		CSF**−** *n* (%) = 8 (40)	CSF+* n* (%) = 12 (60)	Total	*p*
Age (years)	20 (100.0)	Median (IQR)	7.5 (2.9–14.0)	10.5 (6.8–11.5)	10.0 (5.8–13.2)	0.615
Age group	20 (100.0)	1–23 months	1 (12.5)	1 (8.3)	2 (10.0)	1.000
		2–9 years	3 (37.5)	4 (33.3)	7 (35.0)	
		10–18 years	4 (50.0)	7 (58.3)	11 (55.0)	
Gender	20 (100.0)	Male	6 (75.0)	9 (75.0)	15 (75.0)	1.000
		Female	2 (25.0)	3 (25.0)	5 (25.0)	
Seizures	20 (100.0)	No	4 (50.0)	7 (58.3)	11 (55.0)	1.000
		Yes	4 (50.0)	5 (41.7)	9 (45.0)	
Personality/behavioral changes	20 (100.0)	No	6 (75.0)	9 (75.0)	15 (75.0)	1.000
		Yes	2 (25.0)	3 (25.0)	5 (25.0)	
Musculoskeletal symptoms	20 (100.0)	No	4 (50.0)	11 (91.7)	15 (75.0)	0.109
		Yes	4 (50.0)	1 (8.3)	5 (25.0)	
Respiratory symptoms	20 (100.0)	No	7 (87.5)	12 (100.0)	19 (95.0)	0.400
		Yes	1 (12.5)	0 (0.0)	1 (5.0)	
Gastrointestinal symptoms	20 (100.0)	No	5 (62.5)	8 (66.7)	13 (65.0)	1.000
		Yes	3 (37.5)	4 (33.3)	7 (35.0)	
Lymphadenopathy	20 (100.0)	No	7 (87.5)	10 (83.3)	17 (85.0)	1.000
		Yes	1 (12.5)	2 (16.7)	3 (15.0)	
Edema	20 (100.0)	No	7 (87.5)	10 (83.3)	17 (85.0)	1.000
		Yes	1 (12.5)	2 (16.7)	3 (15.0)	
Conjunctival involvement	20 (100.0)	No	7 (87.5)	10 (83.3)	17 (85.0)	1.000
		Yes	1 (12.5)	2 (16.7)	3 (15.0)	
Rash (including hyperpigmentation)	20 (100.0)	No	6 (75.0)	10 (83.3)	16 (80.0)	1.000
		Yes	2 (25.0)	2 (16.7)	4 (20.0)	
Glasgow Coma Scale score	18 (90.0)	Median (IQR)	15.0 (12.0–15.0)	13.0 (10.5–14.2)	13.5 (11.0–15.0)	0.357
Focal neurological deficits^a^	20 (100.0)	No	5 (62.5)	2 (16.7)	7 (35.0)	0.062
		Yes	3 (37.5)	10 (83.3)	13 (65.0)	
Presence of cerebellar signs	20 (100.0)	No	6 (75.0)	8 (66.7)	14 (70.0)	1.000
		Yes	2 (25.0)	4 (33.3)	6 (30.0)	
Signs of meningeal irritation	20 (100.0)	No	5 (62.5)	4 (33.3)	9 (45.0)	0.362
		Yes	3 (37.5)	8 (66.7)	11 (55.0)	
Cranial nerve abnormality	20 (100.0)	No	8 (100.0)	9 (75.0)	17 (85.0)	0.242
		Yes	0 (0.0)	3 (25.0)	3 (15.0)	
Involuntary movements	20 (100.0)	No	7 (87.5)	12 (100.0)	19 (95.0)	0.400
		Yes	1 (12.5)	0 (0.0)	1 (5.0)	
Managed in intensive care unit	20 (100.0)	No	6 (75.0)	5 (41.7)	11 (55.0)	0.197
		Yes	2 (25.0)	7 (58.3)	9 (45.0)	
Duration of hospitalization (days)	19 (95.0)	Median (IQR)	7.0 (6.0–8.2)	8.0 (7.0–10.0)	8.0 (6.5–9.0)	0.242
Liverpool Outcome Score	16 (80)	Poor	2 (25.0)	4 (33.3)	6 (30.0)	0.717
		Good	5 (62.5)	5 (41.7)	10 (50.0)	
Total white blood cell count (×10^9^/L)	20 (100.0)	Median (IQR)	7.7 (6.5–13.3)	10.5 (7.5–13.5)	9.2 (7.0–13.3)	0.537
Absolute lymphocyte count (×10^9^/L)	20 (100.0)	Median (IQR)	3.5 (2.0–5.2)	2.2 (1.9–4.2)	2.4 (1.9–4.9)	0.589
Absolute neutrophil count (×10^9^/L)	20 (100.0)	Median (IQR)	5.0 (3.9–7.3)	6.8 (5.2–10.7)	6.1 (4.5–9.9)	0.280
Platelets count (×10^9^/L)	20 (100.0)	Median (IQR)	216.0 (95.0–343.2)	149.0 (96.8–250.0)	162.5 (96.8–266.5)	0.487
Direct bilirubin (mg/dL)	18 (90)	Median (IQR)	0.2 (0.1–0.3)	0.2 (0.2–1.1)	0.2 (0.1–0.4)	0.467
Aspartate transaminase (AST) (IU/L)	19 (95)	Median (IQR)	44.0 (37.0–63.7)	128.6 (39.8–198.7)	58.0 (39.2–152.0)	0.176
Alanine transaminase (ALT) (IU/L)	19 (95)	Median (IQR)	27.1 (18.8–48.0)	86.4 (47.0–98.2)	56.0 (28.6–95.5)	0.076
Transaminitis (AST/ALT > 80 IU/L)	19 (95)	No	6 (75.0)	3 (25.0)	9 (45.0)	**0.011**
		Yes	1 (12.5)	9 (75.0)	10 (50.0)	
Hepatic dysfunction^b^	20 (100.0)	No	7 (87.5)	6 (50.0)	13 (65.0)	0.158
		Yes	1 (12.5)	6 (50.0)	7 (35.0)	
Urea (mg/dL)	18 (90)	Median (IQR)	29.3 (17.0–38.0)	29.3 (24.8–46.1)	29.3 (22.7–46.1)	0.496
Creatinine (mg/dL)	19 (95)	Median (IQR)	0.6 (0.3–0.7)	0.5 (0.5–0.6)	0.5 (0.4–0.7)	0.804
Sodium (mEq/L)	20 (100.0)	Median (IQR)	138.0 (136.8–141.2)	137.0 (133.8–140.0)	137.0 (135.5–141.2)	0.642
Potassium (mEq/L)	20 (100.0)	Median (IQR)	4.1 (3.7–4.6)	4.3 (4.0–4.9)	4.2 (3.9–4.7)	0.375
Serum albumin (g/dL)	17 (85)	Median (IQR)	3.1 (2.8–3.3)	2.9 (2.6–3.7)	3.0 (2.7–3.6)	0.880
CSF total leukocyte count (cells/µL)	20 (100.0)	Median (IQR)	2.0 (0.8–15.0)	14.5 (6.5–37.5)	7.5 (2.8–36.0)	0.076
CSF lymphocyte count (cells/µL)	20 (100.0)	Median (IQR)	2.0 (0.8–9.0)	7.5 (6.5–35.0)	7.0 (2.8–24.5)	0.063
CSF neutrophil count (cells/µL)	20 (100.0)	Median (IQR)	0.0 (0.0–0.5)	1.5 (0.0–5.5)	0.0 (0.0–4.0)	0.257
CSF pleocytosis	20 (100.0)	No	5 (62.5)	3 (25.0)	8 (40.0)	0.167
		Yes	3 (37.5)	9 (75.0)	12 (60.0)	
CSF protein concentration (mg/dL)	20 (100.0)	Median (IQR)	24.0 (17.7–47.2)	49.2 (39.8–74.3)	40.5 (24.0–62.5)	0.054

*Note:* Categorical variables are represented as no. patients (%) and continuous variables as median (*Q*1–*Q*3).

Abbreviation: *n,* no. of patients in each group.

^a^ Abnormal tone/power/cranial nerve deficits/loss of higher mental function like loss of speech, ability to calculate etc.

^b^ Total bilirubin ≥ 4 mg/dL or ALT two times of upper limit for age.

Clinical and laboratory findings of children with different causes of AES, wherever available, are presented in Table S7.

### 3.2 Amplicon Sequencing of CHIKV

Twelve PCR‐positive samples (four CSF and eight blood) from nine patients (Table 2) underwent amplicon sequencing of CHIKV (Table 4). The Ct values of these samples ranged from 18.5 to 38, with a median Ct value of 35.4. Despite high Ct values, sequencing confirmation was achieved for all PCR‐positive clinical samples. Out of the 12 samples, 9 yielded partial/incomplete genomes (consensus at 1**×**) (Table S8) and 3 yielded near‐complete genomes (consensus generated at 10**×**). Of the 12 sequences, 11 could be classified as belonging to the ECSA genotype. Genotype information for one sequence could not be ascertained due to limited sequence information; however, this sequence was derived from the CSF of a patient whose blood sample was classified as ECSA genotype, indicating all infections with available data were due to the ECSA genotype (Table 4).

**Table 4:** Details of samples subjected to amplicon sequencing.

Patient no.	Sample type	CT value	Total reads	Filtered reads^a^	Mapped reads	% Mapped	Bases covered	% Coverage	Average depth	Genotype assigned
2	CSF	38	78 973	64 002	6	0.01^b^	2450	20.72	0.27	ECSA
	Blood	38	71 416	59496	26	0.04^b^	6585	55.68	2.43	ECSA
3	Blood	36	20 136	13 428	1980	14.75	5459	46.16	59.94	ECSA
4	Blood	33	30 759	20012	8088	40.42	7932	67.07	272.11	ECSA
8	Blood	36	19 454	9828	20	0.20	1533	12.96	0.64	ECSA
13	Blood	37	22 941	11 404	464	4.07	1971	16.67	12.78	ECSA
14	Blood	34.8	33 073	27 890	23 909	85.73	11 790	99.70	705.23	ECSA
15	CSF	23.7	178 351	172 805	157 964	91.41	11 247	95.10	6155.06	ECSA
	Blood	18.5	227 469	226 979	222 902	98.20	11 817	99.92	15085.20	ECSA
18	CSF	34	13 017	2881	24	0.83	556	4.70	0.55	NA
	Blood	37	66 628	53 493	32	0.06^b^	3703	31.31	0.87	ECSA
21	CSF	33.7	13 344	3330	742	22.28	4673	39.51	20.94	ECSA
	Control	—	12469	2758^b^	2	0.07^b^	563	4.76	0.08	ECSA

Abbreviation: NA, this sample had insufficient information to assign a genotype.

^a^ Filtered reads = length > 100 bp, primers removed.

^b^ Low % of reads mapping.

**Figure 1:**
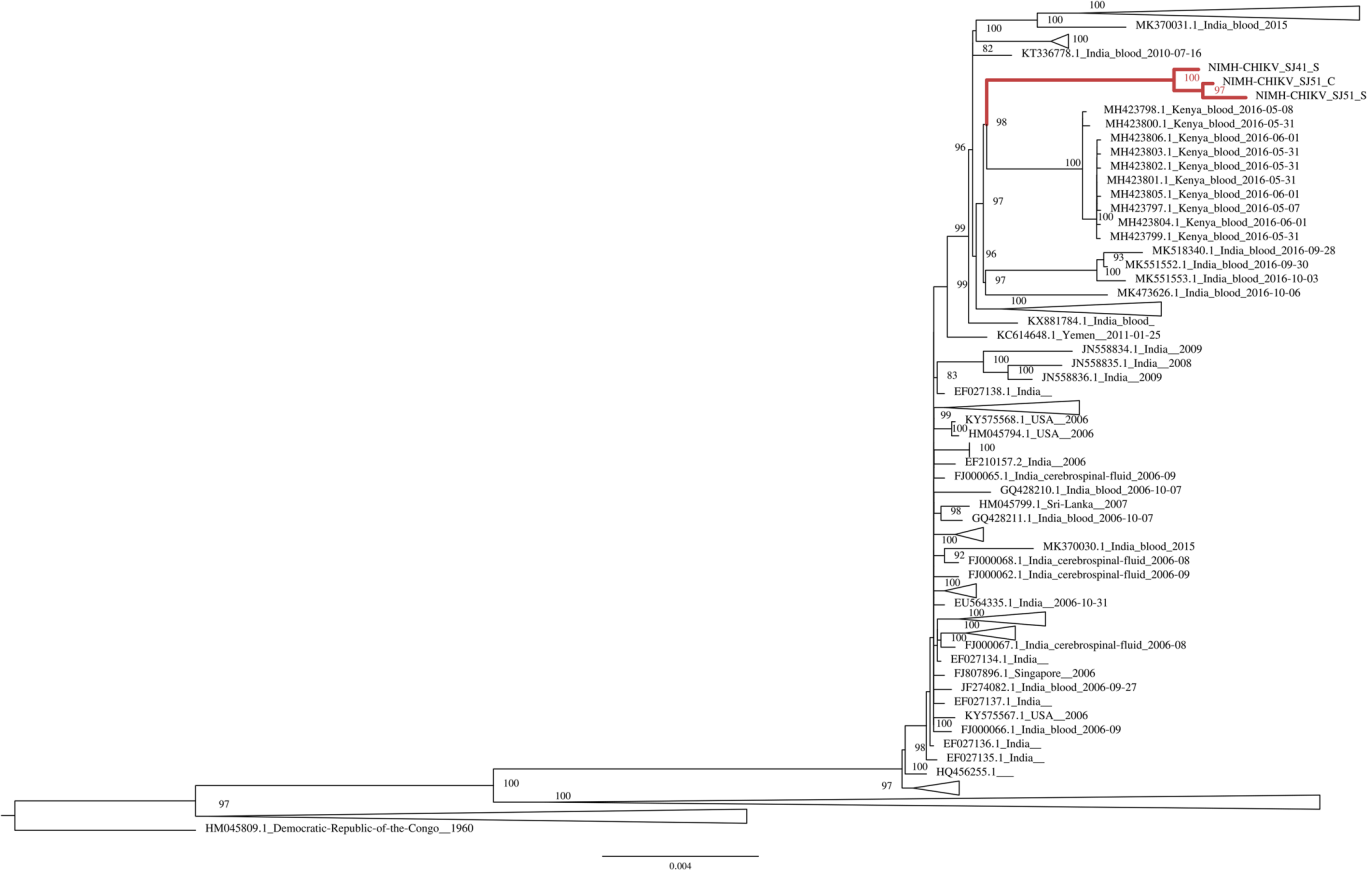
Maximum likelihood phylogenetic tree of CHIKV ECSA genotype (*n *= 461). Figure shows a maximum likelihood tree of CHIKV ECSA sequences based on the region (77–11 313) rooted by the outgroup. Bootstrap support values > 80 are shown. Tips (other than sequences from the study) are labeled as Genbank accession ID of the sequence, country, type of biological sample, and collection date. Branches leading to sequences from the study are highlighted in red. Clades distant from the study sequences have been collapsed.

Among the three near‐complete genomes, two were recovered from the CSF and blood of the same child, and one from the blood of another child. Phylogenetic analysis of global ECSA CHIKV sequences showed that these near‐complete genomes clustered closely with sequences from a 2016 outbreak in Kenya and those circulating in India, forming a separate clade within the ECSA genotype [38, 39] (Figure 1).

### 3.3 Analysis of Envelope Proteins E1 and E2

The CHIKV envelope glycoproteins E1 and E2 from the three near‐complete genomes were compared to the reference sequences NC_004162.2 and S27 (S27 UniProt: Q8JUX5). The in silico translation revealed 25 amino acid substitutions in the CHIKV gp2 amino acid positions 326–1248, spanning the E2 and E1 regions (Table 5). Of these, 21 substitutions have been previously reported in ECSA genomes. Of the four previously unreported/novel amino acid substitutions, S27 E2:M390I, E2:I415V, and E2:I418V were present in all three genomes, while S27 E2:G108R was observed in one of the three sequences. Notably, this mutation was absent in the sequence recovered from CSF of the same patient.

**Table 5:** Predicted amino acid changes in CHIKV envelope glycoprotein, E1 and E2.

			Amino acid			
Region coding for spike glycoprotein	Amino acid position (NC_004162.2)	Amino acid position (S27 UniProt: Q8JUX5)	S27 (UniProt: Q8JUX5)/NC_004162.2	Sequences in the study	No. of sequences with the predicted substitution (*n* = 3)	Present in other ECSA whole genomes
E2	44	57	G	K	3	Yes
Refseq: NC004162.2: 339–742	61	74	I	M	3	Yes
UniProt: Q8JUX5_S27^a^: 326–748	66	79	G	E	3	Yes
	95	108	G	G/R	1	No
	147	160	N	T	3	Yes
	151	164	A	T	3	Yes
	168	181	L	M	3	Yes
	181	194	S	G	3	Yes
	198	211	I	T	3	Yes
	251	264	V	A	3	Yes
	254	267	M	R	3	Yes
	286	299	S	N	3	Yes
	299	312	T	M	3	Yes
	331	344	A	T	3	Yes
	362	375	S	T	3	Yes
	373	386	V	A	3	Yes
	377	390	M	I	3	No
	402	415	I	V	3	No
	—	418	I	V	3	No
E1	13	—	V	I	3	Yes
Refseq: NC004162.2: 744–1247	59	—	I	V	3	Yes
UniProt: Q8JUX5_S27^b^: 810–1248	121	55	I	V	3	Yes^c^
	277	211	K	E	3	Yes
	350	284	D	E	3	Yes
	388	322	V	A	3	Yes

*Note:* As E1 and E2 are part of the polypeptide CHIKV glycoprotein 2 (CHIKV gp2), the amino acid positions from the predicted start of the glycoprotein (as mentioned in brackets as the predicted start and end sites of the E1 and E2) are different for S27 (S27 UniProt: Q8JUX5) and NC_004162.2.

^a^ Uniprot ID: PRO_0000226222.

^b^ Uniprot ID: PRO_0000226224.

^c^ Only present in sequences from India and Kenya 2016.

## 4 Discussion

Microbiological investigations for CHIKV in children with AES in southern India revealed positive test results in 5.3% (20/376) of all AES cases, accounting for 10% (20/193) of those with a microbiological diagnosis, despite the absence of an ongoing CHIKV outbreak.

Comparison with previous studies shows varying prevalence rates of CHIKV in AES cases. During outbreaks, rates ranged from 3% to 14% in different regions of India [14, 23, 40], while studies from Kenya and Cambodia reported 9.2% and 2% of children with CNS infections, respectively [7, 41]. A recent study in Uttar Pradesh, conducted outside an outbreak, found CHIKV in 12/238 (4%) children with AES, like our findings [42].

A striking finding was the high rate of co‐detection or co‐infections of pathogens, with almost three‐fourth of CHIKV‐positive children also testing positive for other pathogens. This mirrors the study from Uttar Pradesh, where all CHIKV‐positive children showed evidence of other pathogens [42].

Importantly, most co‐positive cases showed microbiological evidence of CHIKV infection in the CNS, primarily through the presence of IgM antibodies in CSF. Typically the presence of IgM antibodies in CSF is considered strong evidence of direct CNS involvement by the pathogen, as IgM antibodies do not usually cross the intact blood‐brain barrier (BBB) [43]. However, in the context of co‐positivity with pathogens that are also known to cause neurological infections, such as scrub typhus, which was the most commonly co‐detected pathogen in our study, this interpretation becomes less straightforward. Neurological scrub typhus can induce inflammation and disrupt the BBB, potentially allowing serum anti‐CHIKV IgM antibodies due to recent or past infections, to enter the CSF, even in the absence of direct CHIKV CNS invasion [44, 45, 46]. Second, severe CNS infections can sometimes trigger nonspecific intrathecal antibody production, potentially leading to false‐positive results [47]. Conversely, these findings could represent true co‐infections, as polymicrobial infections, including viral‐bacterial co‐infections, can exacerbate disease severity and alter pathogenesis [48, 49]. The occurrence of even a single case of dual PCR positive for ST and CHIKV in CSF in this study indicates that such co‐infection is at least possible. This complexity underscores the challenges in definitively diagnosing CHIKV‐associated CNS infections in the presence of multiple pathogens.

CHIKV PCR was positive in less than half (9/21) of the children. PCR positivity is known to be optimal when clinical samples are collected within 7 days of symptom onset [30]. In our study, children with positive CHIKV PCR had a median duration of illness of 6.5 days (IQR: 5–9.25) before sample collection, compared to 7.5 days (IQR: 6.5–11.25) for those with negative PCR results. Therefore, relatively late sampling could explain the low rate of PCR positive tests observed in our cohort.

Children with evidence of CHIKV in this study exhibited a wide range of symptoms affecting various organs. In addition to neurological symptoms previously reported [21, 22, 39], we also observed positive cerebellar signs, cranial nerve involvement, involuntary movements, and hemiplegia in these children. Some of these systemic manifestations could be attributed to concurrent co‐infections like scrub typhus, however in five patients CHIKV was the only pathogen identified, highlighting its potential to cause diverse symptoms, as have been reported previously [4, 7, 22, 50]. Additionally, higher transaminase levels in the CSF+ group may suggest the influence of co‐infections with pathogens such as *O. tsutsugamushi* or dengue, or indicate more severe systemic inflammation and multiorgan involvement in this group [50]. The small sample size in this study underscores the need for further research to validate these findings on a larger scale.

Genomic characterization of CHIKV revealed the absence of the S27 E1:A226V but the presence of the S27 E2:I211T amino acid substitutions. Both these mutations were reported in sequences from the large CHIKV epidemic that began on Réunion Island in the Indian Ocean in 2005, which led to CHIKV's adaptation to *A. albopictus*, broadening its vector range and significantly improving its infectivity in *A. albopictus* [37, 51]. Additionally, all three genomes from the study had mutations S27 E1:K211E and S27 E2:V264A, which were first detected in sequences from India in 2008 and have been associated with fitness advantages in the *Aedes aegypti* vector [52, 53]. These mutations were present in sequences from and possibly drove sporadic CHIKV outbreaks in India and neighboring countries between 2010 and 2016 [52, 53, 54]. The coexistence of mutations favouring both *A. aegypti* and *A. albopictus* reflects the presence of both species in India [53].

Sequences from this study also had the S27 E1:D284E and S27 E1:V322E amino acid substitutions, previously reported in genomes from other parts of India, including the south [37, 54, 55]. However our sequences are phylogenetically distinct from other Indian sequences from 2015 to 2017, lacking several predicted amino acid substitutions in E1 and E2 (namely S27 E1:I317V, S27 E1:M269V, S27 E1: K16E/Q, S27 E1: K132Q/T, S27 E1: S355T, S27 E2: C19R and S27 E2:S185Y in the background of S27 E1: K211E and S27 E2:V264A) [54, 56]. Instead, all three isolates had S27 E1:I55V substitution, previously described in genomes from Kenya and India during the 2016 outbreak [38, 39, 52].

Notably, we identified four previously unreported amino acid substitutions in the E2 region, three of which were present in all three sequences (Table 5). The E1 and E2 proteins are crucial for CHIKV's entry into host cells and are primary targets for neutralizing antibodies. Therefore, changes in these proteins could potentially affect virus transmissibility and immune evasion. The S27 E2:G108R mutation, found in only one of the three sequences, may be due to sequencing error and requires further validation.

These novel amino acid changes warrant investigation to understand their potential contribution to increased transmission, disease severity, and neurological manifestations. Overall, these observations signify a shift in the molecular characteristics of circulating CHIKV in southern India, which could have significant implications for public health and necessitate further research.

A key limitation of our study is initial screening using anti‐CHIKV IgM ELISA of serum alone. Incorporating serial blood tests and PCR as first‐line tests, along with confirmation of IgM ELISA by neutralization assays such as plaque reduction neutralization titer (PRNT) measurement, could have improved confirmation rates. However, given the availability and cost‐effectiveness of tests, initial screening with serum IgM ELISA followed by RT‐PCR for confirmation is usually recommended [46, 57]. Additionally, late sample collection and the use of stored nucleic acid that had undergone freeze‐thaw cycles might have impacted the results of PCR tests, sequencing, and genome recovery. Furthermore, the patient numbers and higher rate of co‐positivity presented challenges in accurately assessing CHIKV prevalence in childhood AES. Nevertheless, PCR and sequencing confirmation in nearly half of the patients underscores the significance of CHIKV as a pathogen in AES.

To conclude, our study demonstrates that CHIKV is a significant cause of AES among children in southern India, even outside outbreak periods, emphasizing the need for routine CHIKV testing in this region. This could substantially impact clinical management and public health interventions, particularly given the potential for severe outcomes and the anticipated availability of chikungunya vaccines [58]. The high rate of co‐positivity and the complexity in definitively diagnosing CHIKV‐associated CNS infections in this context highlights the need for comprehensive testing and use of tests beyond routinely used serum IgM ELISA. While the synergistic effects of multiple pathogens in CNS infections are plausible, further research is needed to conclusively establish this relationship. Finally, continued genomic surveillance for CHIKV is crucial to monitor emerging mutations that could impact disease transmission and severity.

## Author Contributions

**Tina Damodar:** conceptualization, data curation, formal analysis, funding acquisition, methodology, project administration, software, visualization, writing–original draft, writing–review and editing. **Chitra Pattabiraman:** conceptualization, data curation, formal analysis, investigation, methodology, writing–original draft, writing– review and editing. **Bhagteshwar Singh:** conceptualization, data curation, writing – review and editing. **Maria Jose:** investigation, methodology, project administration. **Namratha Prabhu:** investigation, methodology, project administration. **Akhila L:** investigation, methodology, project administration. **Pramada Prasad:** investigation. **Uddhava V. Kinhal:** resources, writing–review and editing. **A. V. Lalitha:** resources. **Fulton Sebastian Dsouza:** resources. **Sushma Veeranna Sajjan:** resources. **Vykuntaraju K. Gowda:** resources. **Vasanthapuram Ravi:** conceptualization, methodology, supervision. **Ruwanthi Kolamunnage‐Dona:** formal analysis, writing–review and editing. **Benedict D. Michael:** writing–review and editing. **Tom Solomon:** conceptualization, supervision, writing– review and editing. **Ravi Yadav:** conceptualization, supervision, writing–review and editing. **Lance Turtle:** conceptualization, supervision, writing–original draft, writing–review and editing.

## Conflicts of Interest

Lance Turtle is supported by the National Institute for Health Research Health Protection Research Unit (NIHR HPRU) in Emerging and Zoonotic Infections (NIHR200907) at the University of Liverpool in partnership with Public Health England (PHE), in collaboration with Liverpool School of Tropical Medicine and the University of Oxford. Lance Turtle is based at the University of Liverpool. The views expressed are those of the author(s) and not necessarily those of the NHS, the NIHR, the Department of Health, or Public Health England. Lance Turtle has received consulting fees from MHRA and from AstraZeneca and Synairgen, paid to the University of Liverpool; speakers’ fees from Eisai Ltd; and support for conference attendance from AstraZeneca. Benedict D. Michael is supported to conduct COVID‐19 neuroscience research by the UKRI/MRC (MR/V03605X/1) and by the NIHR Health Protection Research Unit (HPRU) in Emerging and Zoonotic Infections at University of Liverpool. Benedict D. Michael is also supported for additional neurological inflammation research due to viral infection by grants from: the NIHR, the Medical Research Council [MC_PC_19059] the MRC/UKRI (MR/V007181/1), MRC (MR/T028750/1), Wellcome (ISSF201902/3) and Medical Research Foundation (MRF) [MRF‐CPP‐R2‐2022‐100003]. Chitra Pattabiraman and Pramada Prasad were supported by the DBT/Wellcome Trust India Alliance Fellowship (IA/E/15/1/502336) awarded to Chitra Pattabiraman. The other authors declare no conflicts of interest.

## Supporting information

Supporting information.

Supporting information.

Supporting information.

## Data Availability

The data that support the findings of this study are available from the corresponding author upon reasonable request.
