# Supplementary material for: Microbiological Investigations for Chikungunya Virus in Children With Acute Encephalitis Syndrome in a Non‐Outbreak Setting in Southern India
Source: J Med Virol. 2025 Feb 15;97(2):e70233. doi: 10.1002/jmv.70233 (PMC11829551; doi:10.1002/jmv.70233)
Supplement: Supplementary file 1 — Supporting information. [file JMV-97-e70233-s001.docx]

**Supplementary**

**Supplementary Table 1:** Laboratory criteria for determining chikungunya as likely cause of CNS infection

| Microbiological tests for CHIKV | | | | Microbiological tests for other pathogens | | | | CSF Pleocytosis |
| --- | --- | --- | --- | --- | --- | --- | --- | --- |
| Blood | | CSF | | Blood | | CSF | |  |
| Serum IgM ELISA | Blood PCR | CSF IgM ELISA | CSF PCR | Serum IgM ELISA | Blood PCR | CSF IgM ELISA | CSF PCR |  |
| +/- | +/- | +/- | + | +/- | +/- | +/- | +/- | +/- |
| +/- | +/- | + | - | - | - | - | - | +/- |
| +/- | + | - | - | +/- | - | - | - | + |

**Supplementary Table 2:** Primer sequences used for amplicon sequencing

| **Primer Name** | **Sequence (5'-3')** |
| --- | --- |
| CHIK_400_1_LEFT_3 | CTTTTTGAAGGCCCTGCAACGT |
| CHIK_400_1_RIGHT_3 | ACGGCCATCACCTCTTGTAAGT |
| CHIK_400_2_LEFT_0 | TGTCGGACAGGAAGTACCACTG |
| CHIK_400_2_RIGHT_0 | CTCATCCGCCCAATTTGTCGAG |
| CHIK_400_3_LEFT_0 | GTACTGGGTAGGGTTCGACACA |
| CHIK_400_3_RIGHT_0 | CACACGAAACCACTGTGTCACA |
| CHIK_400_4_LEFT_4 | TCGGTGTTCCATCTAAAGGGCA |
| CHIK_400_4_RIGHT_4 | CTTCATGGTGTTCGTGTTCCGT |
| CHIK_400_5_LEFT_0 | GGTGGGGCTGAACCAGAGAATA |
| CHIK_400_5_RIGHT_0 | TACAAAGCTGTCGAATTCGGCC |
| CHIK_400_6_LEFT_4 | AAGCAGAAAACACACACGGTCT |
| CHIK_400_6_RIGHT_4 | GCGCCTCTCGGAGTCTCTATTA |
| CHIK_400_7_LEFT_0 | TACAGGCAGCACAGGAAGATGT |
| CHIK_400_7_RIGHT_0 | TGGAAGTCTTCGGGCGAAATTG |
| CHIK_400_8_LEFT_1 | GGAGCAAGTGAAGACGTGTACG |
| CHIK_400_8_RIGHT_1 | TATATGGACAAGCGGGGCGAAT |
| CHIK_400_9_LEFT_0 | TGCAAGAAGGAAGAAGCTGCAG |
| CHIK_400_9_RIGHT_0 | AAGGCGATCAAGGCAAGTAACG |
| CHIK_400_10_LEFT_0 | ATCTGCACGTACGGTTGATTCG |
| CHIK_400_10_RIGHT_0 | GGTTTTGTTGAGCCCGTAGTGT |
| CHIK_400_11_LEFT_0 | GGCAAAATGCGCACTACGAATG |
| CHIK_400_11_RIGHT_0 | CCTTCCGTACGCGTTAGGAGTA |
| CHIK_400_12_LEFT_1 | AGGAGTTTACGCAGTTAGGCAAA |
| CHIK_400_12_RIGHT_1 | TTCATTCAGGGCTACTTCGGGT |
| CHIK_400_13_LEFT_0 | TGGTCCCTATCCTCGAAACAGC |
| CHIK_400_13_RIGHT_0 | TCCTGGTAGTCACGCAGATCTG |
| CHIK_400_14_LEFT_0 | TGAGGCAGCGTCCATTCTAGAA |
| CHIK_400_14_RIGHT_0 | ATACCTACCAAGTGTTGCCGGT |
| CHIK_400_15_LEFT_4 | ACCTTGCACTGCCTACTAAGAGA |
| CHIK_400_15_RIGHT_4 | ACATGGTGGTTTCAATGCTCTGG |
| CHIK_400_16_LEFT_0 | TCAGAGCATACGGTTACGCAGA |
| CHIK_400_16_RIGHT_0 | TTTGCGGTTCCTACTGGTGTTG |
| CHIK_400_17_LEFT_0 | GACGGTGTTTGCAAGGCAGTAT |
| CHIK_400_17_RIGHT_0 | TCCATGGCTGTAAAGAGGTGGT |
| CHIK_400_18_LEFT_0 | ACCTCTCCTCTCCACAGGTGTA |
| CHIK_400_18_RIGHT_0 | GCCTCTATTTGCTTTGGCCACA |
| CHIK_400_19_LEFT_0 | AAGGGACACGTTTTCACCAGAC |
| CHIK_400_19_RIGHT_0 | GGAGCATTTGACTTTTTGCACTCC |
| CHIK_400_20_LEFT_0 | CGACTTCGCATGAACCATGTCA |
| CHIK_400_20_RIGHT_0 | GTATGTATCGCCCCGTCGTCTA |
| CHIK_400_21_LEFT_0 | AGTTTGATCTAAGCGCCGATGG |
| CHIK_400_21_RIGHT_0 | AAATGTGATGGGGAACGTCTCG |
| CHIK_400_22_LEFT_0 | CGCGTGACACAGCTATTTCCTT |
| CHIK_400_22_RIGHT_0 | TGGCGTACCGACTTCTGTTGTA |
| CHIK_400_23_LEFT_0 | ACACGGACGACGAGTTATGACT |
| CHIK_400_23_RIGHT_0 | GACAATCGGACGTTGATCGGAG |
| CHIK_400_24_LEFT_0 | TAATGGCAGAGACCCCGAAAGT |
| CHIK_400_24_RIGHT_0 | TGTGTTCTGGAATGGGGAAGGT |
| CHIK_400_25_LEFT_0 | CGAGCGACATTCAATCCGTCAA |
| CHIK_400_25_RIGHT_0 | CGGCAGCAGATTATGGGTTCTT |
| CHIK_400_26_LEFT_3 | GAAGAATTTGCTGCCAGCCCTA |
| CHIK_400_26_RIGHT_3 | GTGCGGCTATAATGGCATCGAA |
| CHIK_400_27_LEFT_0 | TTAGGAGATTGAACGCCGTCCT |
| CHIK_400_27_RIGHT_0 | GTCAGACGATCTTCCAACACCC |
| CHIK_400_28_LEFT_2 | GACAGGTACGCGCTTCAAGTTC |
| CHIK_400_28_RIGHT_2 | CGCTAACGGTTTGCCCAGTTTA |
| CHIK_400_29_LEFT_0 | AGCTCCCTACTTTTGTGGAGGG |
| CHIK_400_29_RIGHT_0 | GACCGCCGTACAAGGTTATGAC |
| CHIK_400_30_LEFT_0 | TAATGTCCATGGCCACCTTTGC |
| CHIK_400_30_RIGHT_0 | TATTCTTCCGATTCTTGCGCGG |
| CHIK_400_31_LEFT_0 | CAACTTGCCCAGCTGATCTCAG |
| CHIK_400_31_RIGHT_0 | ATCGATGGTCCCCTTTACGTGT |
| CHIK_400_32_LEFT_0 | TAAGGTAACAGGTTACGCGTGC |
| CHIK_400_32_RIGHT_0 | TACGGGCTCCTTCATTAGCTCC |
| CHIK_400_33_LEFT_2 | GACCGATCTTCGACAACAAGGG |
| CHIK_400_33_RIGHT_2 | GCGTCGGGGAGAACATGTTAAG |
| CHIK_400_34_LEFT_0 | GAGAAAACCTTGCGCATGCTTG |
| CHIK_400_34_RIGHT_0 | CTGCTGGCATGTGATTGTCCAT |
| CHIK_400_35_LEFT_0 | CCAGGTTTCCTTGCAAATCGGA |
| CHIK_400_35_RIGHT_0 | GTACGTGCTGCAAGGTAGTTCC |
| CHIK_400_36_LEFT_1 | CATGTACGCACCCATTTCACCA |
| CHIK_400_36_RIGHT_1 | GGTCCCCGAATTCAGCATTACG |
| CHIK_400_37_LEFT_0 | CTGCAAGGTCGATCAATGCCAT |
| CHIK_400_37_RIGHT_0 | TCATTGTTACCCCACGTGACCT |
| CHIK_400_38_LEFT_0 | AGTGGGTGACGCATAAGAAGGA |
| CHIK_400_38_RIGHT_0 | TGGTGTCAGTTCGTACGGTGTA |
| CHIK_400_39_LEFT_0 | ATACTCCTGTCGATGGTGGGTG |
| CHIK_400_39_RIGHT_0 | CGGGATCACTGTTACGTGTTCG |
| CHIK_400_40_LEFT_0 | TTTTTAGCCGTACTGAGCGTCG |
| CHIK_400_40_RIGHT_0 | ATTTTCGGTGTCGCAGAAGCAG |
| CHIK_400_41_LEFT_0 | GCGGTACAGCAGAGTGTAAGGA |
| CHIK_400_41_RIGHT_0 | GTCGCCTTTGTACACCACGATT |
| CHIK_400_42_LEFT_0 | CCATGCCGTCACAGTTAAGGAC |
| CHIK_400_42_RIGHT_0 | AGTTCATCGCTCTTACCGGGTT |
| CHIK_400_43_LEFT_0 | ATTGGCTAAAAGAACGAGGGGC |
| CHIK_400_43_RIGHT_0 | TTCCCGAATAGTGACGGCGTTA |
| CHIK_400_44_LEFT_0 | GCACCCATTCCTCAGACTTTGG |
| CHIK_400_44_RIGHT_0 | CCTGCTAAACGACACGCATAGC |
| chikv_11,806 R | ACATCTCCTACGTCCCTGTG |
| chikv_11,209 F | GCGATGTCATGGGTGCAGAA |
| chikv_394 R | TTTTCCTGTGGCAGATGCTA |
| chikv_11 F | GAGACACACGTAGCCTACCAG |

**Supplementary Table 3:** Aetiological distribution of children with AES (n=376)

| Aetiology | No. patients | % |
| --- | --- | --- |
| Chikungunya* | 20 | 5.3 |
| *Orientia tsutsugamushi* | 75 | 19.9 |
| Dengue | 10 | 2.7 |
| JEV | 19 | 5.1 |
| *Leptospira* sp | 8 | 2.1 |
| Herpes Simplex Virus (HSV)-1 & 2 | 4 | 1.1 |
| Mumps | 2 | 0.5 |
| Measles | 2 | 0.5 |
| Others# | 8 | 2.1 |
| Multiple possible aetiologies | 45 | 12.0 |
| Unknown | 183 | 48.7 |
| Total | 376 | 100.0 |

*All cases where test for chikungunya were positive (including those with microbiological evidence of other pathogens)

#Results based on tests performed in the collaborating centres from where children were recruited- includes *Rickettsia* sp (n=4), *Mycobacterium tuberculosis* (n=2), *Salmonella typhi* (n=1), *Methicillin Resistant Staphylococcus aureus* (n=1)

**Supplementary Table 4:** Tests for CHIKV in children with microbiological evidence of other pathogens (n=16)

| **Blood** | | **CSF** | | **No. patients (%)** |
| --- | --- | --- | --- | --- |
| **Serum IgM ELISA** | **Blood PCR** | **CSF IgM ELISA** | **CSF PCR** |  |
| + | + | + | + | 1 (6) |
| + | + | - | + | 1 (6) |
| + | + | - | - | 4 (25) |
| + | - | + | - | 7 (44) |
| + | - | - | - | 3 (19) |

**Supplementary Table 5: Clinical presentations, neurological findings and outcomes of children with CHIKV-associated AES**

| **Patient no.** | **Age/ Gender** | **Clinical presentation** | **General examination findings** | **Neurological findings** | **LOS*** |
| --- | --- | --- | --- | --- | --- |
| 1 | 7 years/ Female | Fever, abnormal speech, vomiting | Periorbital oedema | Meningeal irritation | 3 |
| 2 | 11 years/ Male | Fever, seizures, abnormal speech, irritability, behavioural change, vomiting, abdominal pain | Lymphadenopathy | Meningeal irritation, Abnormal plantar reflex | 3 |
| 3 | 5 years/ Male | Fever, irritability, joint pain, abdominal distension | Lymphadenopathy | Meningeal irritation, Involuntary movement (lip smacking), Cerebellar sign(s) present | 5 |
| 4 | 10 years/ Male | Fever, behavioural change, vomiting, muscle pain, abdominal distension |  | Left hemiparesis, Cerebellar sign(s) present | 3 |
| 5 | 11 years/ Female | Fever, seizures (absence), irritability, vomiting, | Lower limb oedema, conjunctival congestion | 6th cranial nerve involvement, meningeal irritation | 2 |
| 6 | 3 years/ Male | Fever, seizures (GTCS), irritability, muscle pain | Conjunctival congestion |  | 5 |
| 7 | 13 years/ Male | Fever, change in behaviour, abnormal speech |  | Meningeal irritation, Cerebellar sign(s) present | Could not be assessed |
| 8 | 8 years/ Male | Fever, seizures (absence), change in behaviour, irrelevant talk, vomiting | Conjunctival congestion |  | 5 |
| 9 | 10 years/ Male | Fever, change in behaviour, abnormal speech, vomiting | Lymphadenopathy | Meningeal irritation, Cerebellar sign(s) present | 3 |
| 10 | 15 years/ Female | Fever, seizures (GTCS), vomiting, irritability |  |  | 4 |
| 11 | 2.5 years/ Male | Fever, seizures (GTCS), irritability |  | Meningeal irritation | 3 |
| 12 | 15 years/ Male | Fever, abnormal speech |  | Meningeal irritation, 6th & 7th cranial nerve involvement, Cerebellar sign(s) present, sluggish deep tendon reflexes, hypotonia | 5 |
| 13 | 1 year/ Female | Fever, seizures (GTCS), drowsiness |  |  | 5 |
| 14 | 17 years/ Male | Fever, irrelevant talk |  |  | 5 |
| 15 | 11 years/ male | Fever, abnormal speech, joint and muscle pain, abdominal distension | Rash | Meningeal irritation, Cerebellar sign(s) present | Could not be assessed |
| 16 | 6 years/ Male | Fever, vomiting, drowsiness, abdominal pain and distention |  | Meningeal irritation | 5 |
| 17 | 6 years/ Male | Fever, seizures (GTCS), irritability |  | Left hemiplegia, 7th cranial nerve involvement | 5 |
| 18 | 2 months/ Male | Fever, diarrhoea, lethargy | Generalized skin hyperpigmentation |  | Could not be assessed |
| 19 | 14 years/ Male | Fever, seizures (absence), abnormal speech |  | Meningeal irritation | Could not be assessed |
| 20 | 14 years/ Female | Fever, change in behaviour, irrelevant talk, vomiting, cough, joint and muscle pain, abdominal pain, | Facial and Lower limb oedema , Rash, Icterus, healed axillary eschar |  | 5 |
| 21 | 5 years/ Female** | Fever, seizures (multiple staring episodes with teeth clenching), drowsiness, recurrent respiratory tract infection, failure to gain weight | Triangular facies, hypopigmented hair, pallor, severe acute malnutrition, bilateral coarse  crepitations and hepatosplenomegaly |  | Could not be assessed |

*LOS: Liverpool Outcome Score (LOS): 5 = Full recovery. 4 = Minor sequelae with no effect, or only minor effects, on physical function; or personality change; or on medication, 3 = Moderate sequelae mildly affecting function, probably compatible with independent living, 2 = Severe sequelae, impairing function sufficient to make patient dependent

**Child with primary immunodeficiency disease

**Supplementary Table 6: Patient-wise brain imaging findings (MR/ CT scan)**

| **S.No** | **Patient No.** | **Likely cause of CNS infection** | **Findings** |
| --- | --- | --- | --- |
| 1 | 8 | CHIKV | Diffuse, asymmetric involvement of sub-cortical white matter, deep grey matter and cerebellar white matter suggestive of acute viral encephalitis. |
| 2 | 12 | CHIKV | Signs of meningitis with possible cerebellitis |
| 3 | 14 | CHIKV | Not performed |
| 4 | 18 | CHIKV | Not performed |
| 5 | 15 | CHIKV | Normal picture |
| 6 | 21* | CHIKV | Signs of meningo-encephalitis |
| 7 | 2 | CHIKV and Scrub typhus | Not performed |
| 8 | 4 | Unclear/ CHIKV | Not performed |
| 9 | 13 | Unclear/ CHIKV | Normal picture |
| 10 | 17 | Unclear/ CHIKV | Not performed |
| 11 | 19 | Unclear/ CHIKV | Normal picture |
| 12 | 1 | Scrub typhus | Not performed |
| 13 | 9 | Scrub typhus | Not performed |
| 14 | 16 | Scrub typhus | Normal picture |
| 15 | 20 | Unclear/ Scrub typhus | Normal picture |
| 16 | 3 | Unclear | Not performed |
| 17 | 5 | Unclear | Normal picture |
| 18 | 6 | Unclear | Not performed |
| 19 | 7 | Unclear | Normal picture |
| 20 | 10 | Unclear | Not performed |
| 21 | 11 | Unclear | Bilateral hyperintensities in bilateral thalamus, pons and left cerebellar and left frontal sulcus. Features suggestive of viral hemorrhagic encephalitis |

*Child with primary immunodeficiency disease excluded from the study

**Supplementary Table 7**: Clinical and laboratory variables of children with different causes of AES*

| **Clinical Variables** | **No patients (%)** |  | **Chikungunya**  **(n=8)** | **Scrub typhus**  **(n=75)** | **Dengue**  **(n=10)** | **JEV**  **(n=19)** | **Leptospira**  **(n=8)** | **Others**  **(n=18)** | **Multiple positive**  **(n=23)** | **Unknown**  **(n=161)** |
| --- | --- | --- | --- | --- | --- | --- | --- | --- | --- | --- |
| Age (years) | 322 (100) | Median (IQR) | 10.5 (6.2 to 14.2) | 8.0 (4.0 to 10.5) | 4.0 (1.6 to 6.8) | 7.0 (4.0 to 12.5) | 10.5 (6.0 to 12.5) | 5.5 (1.1 to 14.8) | 6.0 (3.5 to 10.0) | 3.0 (1.2 to 9.5) |
| Gender | 322 (100) | Male | 7 (87.5) | 47 (62.7) | 6 (60.0) | 12 (63.2) | 4 (50.0) | 12 (66.7) | 12 (52.2) | 92 (57.1) |
|  |  | Female | 1 (12.5) | 28 (37.3) | 4 (40.0) | 7 (36.8) | 4 (50.0) | 6 (33.3) | 11 (47.8) | 69 (42.9) |
| Seizures | 322 (100) | No | 5 (62.5) | 27 (36.0) | 0 (0.0) | 4 (21.1) | 5 (62.5) | 9 (50.0) | 8 (34.8) | 40 (24.8) |
|  |  | Yes | 3 (37.5) | 48 (64.0) | 10 (100.0) | 15 (78.9) | 3 (37.5) | 9 (50.0) | 15 (65.2) | 121 (75.2) |
| Musculoskeletal symptoms | 322 (100) | No | 6 (75.0) | 68 (90.7) | 10 (100.0) | 17 (89.5) | 7 (87.5) | 16 (88.9) | 22 (95.7) | 155 (96.3) |
|  |  | Yes | 2 (25.0) | 7 (9.3) | 0 (0.0) | 2 (10.5) | 1 (12.5) | 2 (11.1) | 1 (4.3) | 6 (3.7) |
| Lymphadenopathy | 322 (100) | No | 8 (100.0) | 61 (81.3) | 9 (90.0) | 19 (100.0) | 8 (100.0) | 17 (94.4) | 22 (95.7) | 156 (96.9) |
|  |  | Yes | 0 (0.0) | 14 (18.7) | 1 (10.0) | 0 (0.0) | 0 (0.0) | 1 (5.6) | 1 (4.3) | 5 (3.1) |
| Oedema | 322 (100) | No | 8 (100.0) | 58 (77.3) | 8 (80.0) | 17 (89.5) | 7 (87.5) | 17 (94.4) | 19 (82.6) | 148 (91.9) |
|  |  | Yes | 0 (0.0) | 17 (22.7) | 2 (20.0) | 2 (10.5) | 1 (12.5) | 1 (5.6) | 4 (17.4) | 13 (8.1) |
| Conjunctival involvement | 322 (100) | No | 7 (87.5) | 61 (81.3) | 10 (100.0) | 19 (100.0) | 8 (100.0) | 17 (94.4) | 22 (95.7) | 152 (94.4) |
|  |  | Yes | 1 (12.5) | 14 (18.7) | 0 (0.0) | 0 (0.0) | 0 (0.0) | 1 (5.6) | 1 (4.3) | 9 (5.6) |
| Rash | 322 (100) | No | 7 (87.5) | 58 (77.3) | 8 (80.0) | 17 (89.5) | 6 (75.0) | 13 (72.2) | 22 (95.7) | 137 (85.1) |
|  |  | Yes | 1 (12.5) | 17 (22.7) | 2 (20.0) | 2 (10.5) | 2 (25.0) | 5 (27.8) | 1 (4.3) | 24 (14.9) |
| Presence of cerebellar signs | 322 (100) | No | 5 (62.5) | 65 (86.7) | 10 (100.0) | 17 (89.5) | 8 (100.0) | 17 (94.4) | 19 (82.6) | 151 (93.8) |
|  |  | Yes | 3 (37.5) | 10 (13.3) | 0 (0.0) | 2 (10.5) | 0 (0.0) | 1 (5.6) | 4 (17.4) | 10 (6.2) |
| Signs of meningeal irritation | 322 (100) | No | 5 (62.5) | 44 (58.7) | 10 (100.0) | 12 (63.2) | 4 (50.0) | 11 (61.1) | 14 (60.9) | 112 (69.6) |
|  |  | Yes | 3 (37.5) | 31 (41.3) | 0 (0.0) | 7 (36.8) | 4 (50.0) | 7 (38.9) | 9 (39.1) | 49 (30.4) |
| Cranial Nerve Abnormality | 322 (100) | No | 7 (87.5) | 68 (90.7) | 10 (100.0) | 17 (89.5) | 6 (75.0) | 17 (94.4) | 20 (87.0) | 147 (91.3) |
|  |  | Yes | 1 (12.5) | 7 (9.3) | 0 (0.0) | 2 (10.5) | 2 (25.0) | 1 (5.6) | 3 (13.0) | 14 (8.7) |
| **Laboratory Variables** | **No patients (%)** |  | **Chikungunya**  **(n=8)** | **Scrub typhus**  **(n=75)** | **Dengue**  **(n=10)** | **JEV**  **(n=19)** | **Leptospira**  **(n=8)** | **Others**  **(n=18)** | **Multiple positive**  **(n=23)** | **Unknown**  **(n=161)** |
| Total white blood cell count (× 10^9^/L) | 321 (99.7) | Median (IQR) | 7.5 (6.9 to 14.3) | 12.0 (8.8 to 14.6) | 10.6 (8.8 to 15.4) | 11.4 (6.9 to 12.9) | 11.4 (8.3 to 15.8) | 10.1 (9.0 to 14.5) | 7.9 (4.8 to 12.1) | 10.8 (7.7 to 15.8) |
| Absolute Lymphocyte Count (× 10^9^/L) | 318 (98.8) | Median (IQR) | 2.1 (1.3 to 4.4) | 4.1 (2.5 to 5.3) | 3.3 (2.2 to 5.2) | 2.7 (1.1 to 4.2) | 1.6 (1.0 to 4.6) | 2.5 (1.7 to 3.9) | 1.8 (1.3 to 2.7) | 2.4 (1.5 to 4.5) |
| Absolute Neutrophil Count (× 10^9^/L) | 318 (98.8) | Median (IQR) | 5.6 (4.8 to 8.3) | 6.6 (4.5 to 9.2) | 6.4 (4.2 to 9.1) | 7.0 (4.8 to 10.0) | 9.5 (4.8 to 11.0) | 6.5 (5.2 to 8.2) | 4.2 (2.8 to 8.8) | 7.1 (4.1 to 11.1) |
| Platelets count (× 10^9^/L) | 321 (99.7) | Median (IQR) | 254.0 (148.5 to 459.5) | 123.0 (69.0 to 201.0) | 92.5 (62.2 to 301.8) | 335.0 (190.0 to 383.0) | 153.0 (125.0 to 231.5) | 297.0 (184.8 to 397.8) | 176.0 (108.0 to 200.0) | 276.0 (155.5 to 372.0) |
| Direct Bilirubin (mg/dL) | 275 (85.4) | Median (IQR) | 0.1 (0.1 to 0.2) | 0.3 (0.1 to 1.6) | 0.2 (0.2 to 0.5) | 0.1 (0.1 to 0.2) | 0.3 (0.2 to 0.6) | 0.2 (0.1 to 0.6) | 0.1 (0.1 to 0.2) | 0.1 (0.1 to 0.3) |
| Aspartate transaminase (AST) (IU/L) | 308 (95.7) | Median (IQR) | 39.5 (24.1 to 42.0) | 87.9 (59.3 to 206.2) | 122.9 (68.0 to 1122.8) | 31.3 (26.0 to 37.4) | 62.9 (22.5 to 93.8) | 31.4 (18.2 to 81.7) | 52.2 (33.9 to 91.5) | 45.2 (30.7 to 74.6) |
| Alanine transaminase (ALT) (IU/L) | 308 (95.7) | Median (IQR) | 40.0 (18.8 to 67.9) | 62.8 (39.6 to 129.2) | 136.4 (33.1 to 845.0) | 21.5 (13.4 to 40.1) | 44.1 (38.2 to 66.8) | 18.3 (9.5 to 47.5) | 29.0 (19.6 to 56.3) | 22.2 (13.5 to 42.4) |
| Urea (mg/dL) | 286 (88.8) | Median (IQR) | 29.3 (26.9 to 45.4) | 25.4 (18.0 to 33.4) | 38.6 (30.5 to 48.1) | 27.0 (23.2 to 34.2) | 22.5 (21.1 to 37.7) | 22.8 (18.6 to 30.1) | 18.0 (13.4 to 29.9) | 23.0 (17.0 to 35.5) |
| Creatinine (mg/dL) | 314 (97.5) | Median (IQR) | 0.7 (0.7 to 0.8) | 0.4 (0.3 to 0.5) | 0.5 (0.3 to 0.6) | 0.5 (0.4 to 0.7) | 0.6 (0.5 to 0.6) | 0.6 (0.4 to 0.7) | 0.4 (0.3 to 0.5) | 0.4 (0.3 to 0.5) |
| Serum albumin (g/dL) | 279 (86.6) | Median (IQR) | 4.0 (3.6 to 4.5) | 2.8 (2.5 to 3.1) | 3.0 (2.8 to 3.8) | 3.8 (3.1 to 4.3) | 3.7 (2.9 to 3.8) | 3.0 (2.7 to 3.6) | 3.5 (3.0 to 3.8) | 3.7 (3.1 to 4.2) |
| CSF total leucocyte count (cells/µL) | 305 (94.7) | Median (IQR) | 5.0 (2.5 to 8.5) | 10.0 (3.8 to 45.0) | 0.0 (0.0 to 5.0) | 3.5 (2.0 to 8.8) | 30.0 (0.8 to 57.5) | 15.0 (4.0 to 30.0) | 5.0 (1.0 to 21.0) | 2.0 (0.0 to 15.5) |
| CSF lymphocyte count (cells/µL) | 305 (94.7) | Median (IQR) | 5.0 (2.5 to 7.2) | 10.0 (3.0 to 32.2) | 0.0 (0.0 to 5.0) | 3.5 (2.0 to 6.0) | 8.5 (0.8 to 48.8) | 15.0 (4.0 to 30.0) | 5.0 (1.0 to 21.0) | 2.0 (0.0 to 11.2) |
| CSF neutrophil count (cells/µL) | 305 (94.7) | Median (IQR) | 0.0 (0.0 to 0.8) | 0.0 (0.0 to 2.0) | 0.0 (0.0 to 0.0) | 0.0 (0.0 to 0.0) | 0.0 (0.0 to 6.2) | 0.0 (0.0 to 5.0) | 0.0 (0.0 to 0.0) | 0.0 (0.0 to 0.0) |
| CSF protein concentration (mg/dL) | 310 (96.3) | Median (IQR) | 31.5 (17.7 to 40.2) | 57.1 (43.5 to 94.0) | 20.0 (15.0 to 22.0) | 31.4 (20.7 to 46.0) | 53.3 (31.2 to 62.9) | 51.9 (21.9 to 65.0) | 32.3 (19.6 to 42.9) | 28.7 (17.6 to 48.0) |

*Includes PCR-confirmed cases (without serological evidence of an alternate pathogen in CSF) and cases with serological evidence (IgM ELISA+) in the absence of evidence of an alternate aetiology

Categorical variables are represented as no. patients (%) and continuous variables as Median (Q1 to Q3); n= no. of patients in each group.

**Supplementary Table 8:** Coverage data of partial and full genomes

| **Patient No.** | **CSF** | **Blood** |
| --- | --- | --- |
| 2 | 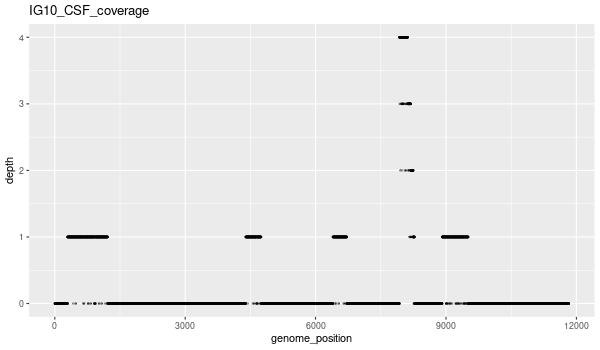 | 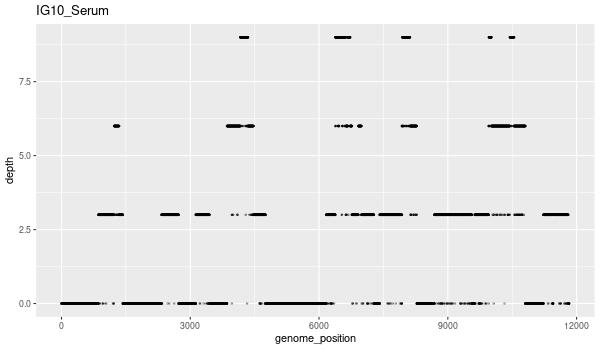 |
| 3 |  | 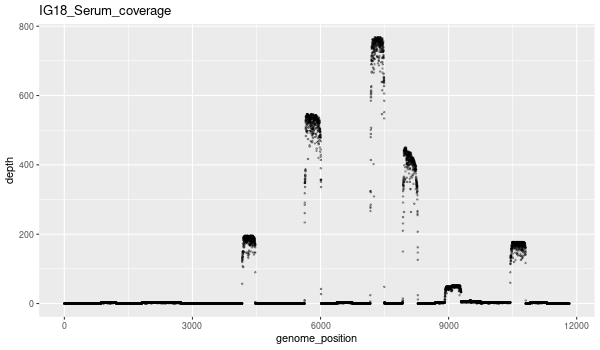 |
| 4 |  | 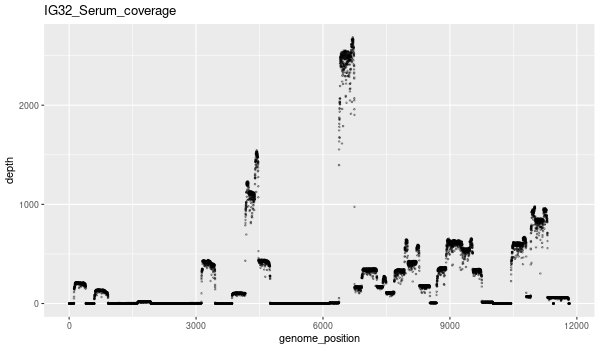 |
| 8 |  | 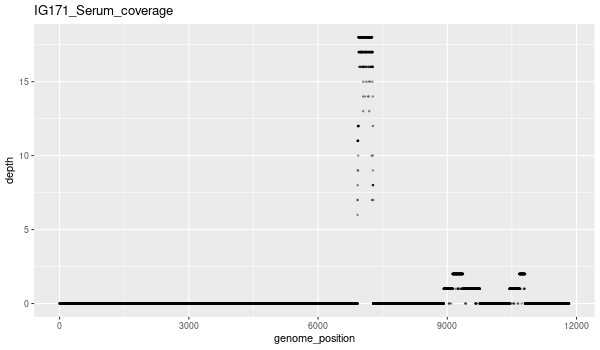 |
| 13 |  | 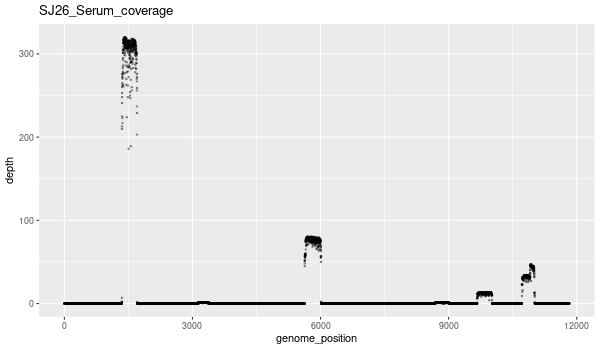 |
| 14 |  | 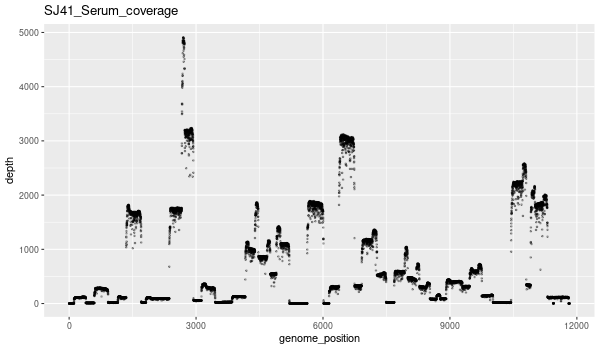 |
| 15 | 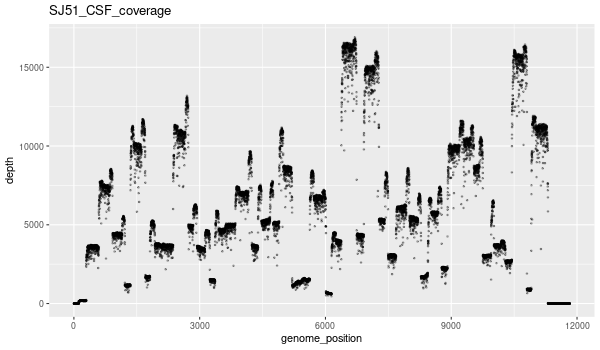 | 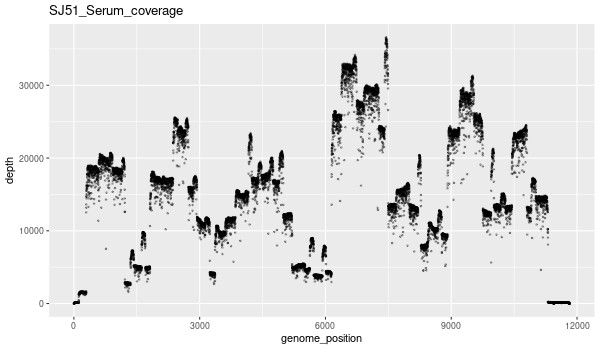 |
| 18 | 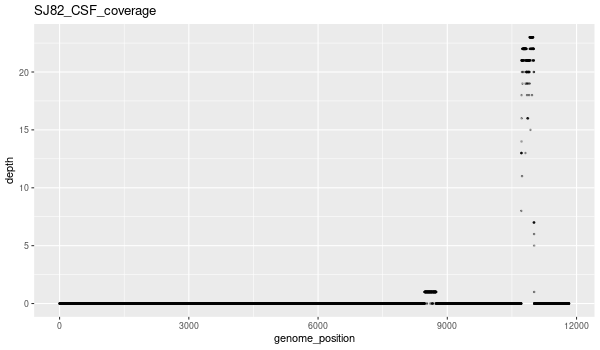 | 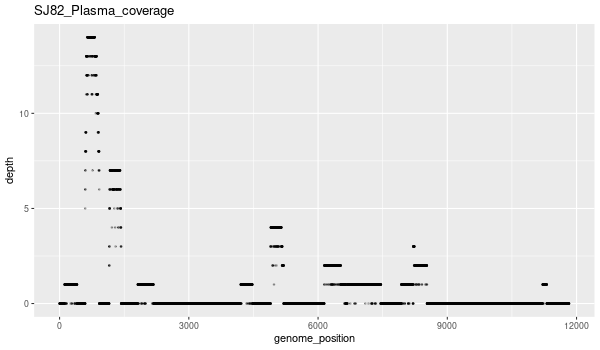 |
| 21 | 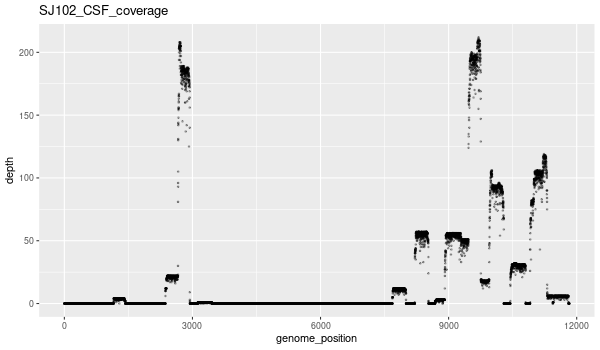 |  |
| Control | 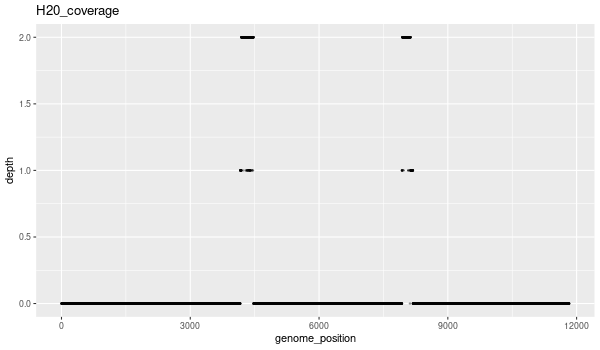 |  |

**Supplementary Figure 1:** Recruitment strategy of patients in the study

**Supplementary Figure 2:** Month-wise distribution of AES and CHIKV positivity
