# Supplementary figures and images for: Microbiological Investigations for Chikungunya Virus in Children With Acute Encephalitis Syndrome in a Non‐Outbreak Setting in Southern India
Source: J Med Virol. 2025 Feb 15;97(2):e70233. doi: 10.1002/jmv.70233 (PMC11829551; doi:10.1002/jmv.70233)

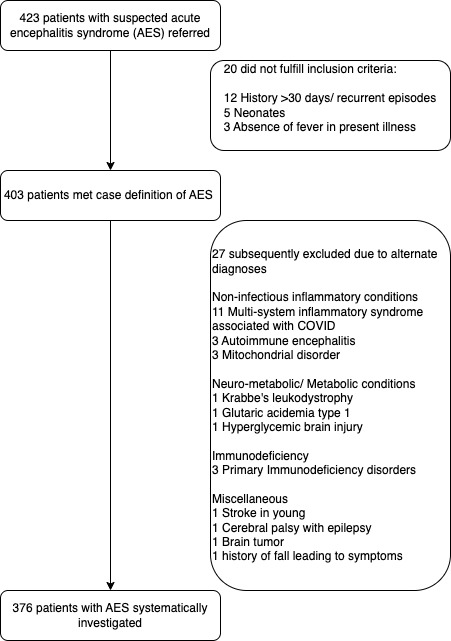

Supplement: Supplementary file 2 — Supporting information. [file JMV-97-e70233-s003.jpg]

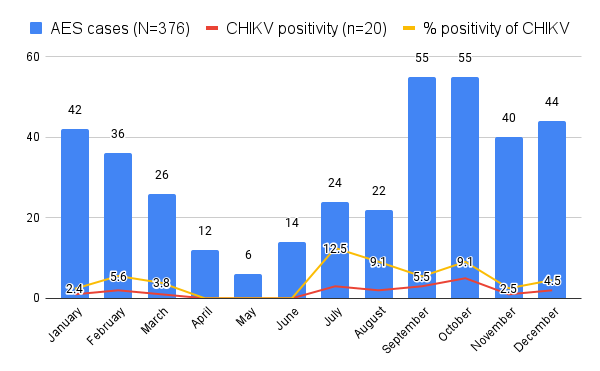

Supplement: Supplementary file 3 — Supporting information. [file JMV-97-e70233-s002.png]
